# Association between hemoglobin glycation index and low bone mineral density in postmenopausal women: An analysis of data from the NHANES database

**DOI:** 10.1097/MD.0000000000044771

**Published:** 2025-09-26

**Authors:** Yuchen Wang, Weizhong Yu, Wenke Zhu, Chuan Jia, Changjun Yun

**Affiliations:** a Department of Orthopedics, Wujin Hospital Affiliated with Jiangsu University, Changhzou, PR China; b Department of Orthopedics, The Wujin Clinical college of Xuzhou Medical University, Changhzou, PR China; c Department of Orthopedics, Changzhou Wujin Traditional Chinese Medicine Hospital, Changhzou, PR China.

**Keywords:** bone mineral density, dietary antioxidant quality score, hemoglobin glycation index, postmenopausal women, risk

## Abstract

The hemoglobin glycation index (HGI), a valuable indicator reflecting individual variations in glycosylated hemoglobin (HbA1c), serves as a crucial measure of glucose metabolism, which may influence bone health. This study aimed to elucidate the association between HGI and low bone mineral density (BMD) in postmenopausal women. Utilizing data from the National Health and Nutrition Examination Survey database across the years 2005 to 2010, 2013 to 2014, and 2017 to 2018, we defined low BMD as a *T*-score ≤ −1.0 and employed logistic regression models to investigate the HGI-BMD relationship, further stratifying the analysis based on dietary antioxidant quality score (DAQS) classifications. From 1961 postmenopausal women included in our analysis, 1197 (60.56%) exhibited low BMD, with results indicating that women with HGI ≥ −0.0835 had elevated odds of low BMD (odds ratio [OR] = 1.47, 95% confidence interval [CI]: 1.09–1.98) compared to their counterparts with HGI < −0.0835. Notably, this association manifested significantly in participants with DAQSs <5 (OR = 1.44, 95% CI: 1.00–2.07, *P* = .049) while remaining absent among those with higher DAQSs. Subgroup analyses revealed that the increased odds of low BMD were predominant in women aged ≥65 years (OR = 1.80, 95% CI: 1.29–2.52) and those without a history of fractures (OR = 1.57, 95% CI: 1.16–2.13). Consequently, our findings endorse the hypothesis that elevated HGI correlates with a greater risk of low BMD in postmenopausal women, a relationship potentially modulated by dietary antioxidant intake, highlighting the need for further exploration of interventions targeting metabolic pathways influencing bone density.

## 1. Introduction

Low bone mineral density (BMD) is a common metabolic bone disease such as osteopenia, which further progresses to osteoporosis, leading to increased bone fragility and fracture risk.^[1]^ The prevalence of low BMD is very common worldwide, with a global prevalence of 19.7% for osteoporosis and 40.4% for osteopenia.^[2]^ In addition, low BMD is highly prevalent in postmenopausal women, and one-third of women over the age of 50 suffer from osteopenia, which is an important cause of fractures.^[3,4]^ Low BMD causes a greater disease burden and severely affects the quality of life and long-term health of patients.^[5,6]^

Glycated hemoglobin (HbA1c) is a commonly used indicator to reflect the long-term change and control of blood glucose, which is associated with the development of many chronic diseases, such as type 2 diabetes mellitus, chronic kidney disease (CKD), and cardiovascular disease.^[7–9]^ Studies have suggested that high HbA1c levels may be a risk factor for fractures,^[10]^ but conclusions about the effect of HbA1c on osteoporosis and BMD are controversial.^[11–14]^ These inconsistent findings may be related to the fact that HbA1c is affected by a variety of factors, including blood glucose levels. Hemoglobin Glycation Index (HGI), which is calculated by the difference between measured and predicted values of HbA1c, quantifies the mismatch between HbA1c and an individual’s average blood glucose,^[15]^ allowing for better identification of interindividual differences in HbA1c at the same glucose level.^[16,17]^ Previous studies have shown that HGI is strongly associated with the risk of cardiovascular events and CKD in both diabetic and nondiabetic populations.^[18–20]^ However, the association between HGI and low BMD remains unclear. In addition, oxidative stress/inflammation and advanced glycosylation end products may be involved in the effects of glucose metabolism on BMD,^[21,22]^ and dietary antioxidant nutrients have an ameliorative effect on these pathological conditions.^[23,24]^ However, whether dietary antioxidants affect the relationship between blood glucose and BMD remains to be explored. A dietary antioxidant quality score (DAQS) calculated based on the intake of multiple antioxidant vitamins and minerals has been proposed and found to be associated with the risk of death in diabetic patients.^[25]^ Thus, this study aimed to investigate the association between HGI and low BMD in postmenopausal women and to further analyze the impact of DAQSs on this association.

## 2. Materials and methods

### 2.1. Data source and study design

Data for this cross-sectional study were obtained from the National Health and Nutrition Examination Survey (NHANES) database. NHANES is a cross-sectional survey conducted every 2 years by the National Center for Health Statistics to assess the health and nutrition status of the US population (https://wwwn.cdc.gov/nchs/nhanes/AnalyticGuidelines.aspx). NHANES uses a complex multi-stage probability sampling design to select the survey population and collects data from participants through interviews and physical examinations. Data derived from interviews included demographic, socioeconomic, dietary, and health-related questions, data derived from physical examinations included medical, dental, and physiological measurements, as well as laboratory tests. Because complete BMD data for total femur and femoral neck were available for only a few survey cycles, the analysis in this study only used data from 2005 to 2006, 2007 to 2008, 2009 to 2010, 2013 to 2014, and 2017 to 2018 NHANES survey cycles. Postmenopausal women aged 40 years or older were included in this study. The exclusion criteria were as follows: women without measurement of HbA1c and blood glucose; women without measurement of BMD; and women without complete for calculating DAQS (intake of vitamin A, vitamin E, vitamin C, zinc, magnesium, and selenium). Participants in NHANES were provided written informed consent. Because the data for this study were derived from de-identified data from publicly available databases (NHANES), this study was exempted from review by the Changzhou Wujin Traditional Chinese Medicine Hospital Ethics Institutional Committee.

### 2.2. Assessment of BMD

The outcome variable was low BMD (*T*-score ≤ −1.0). Participants with a *T*-score ≤ −1.0 for total femur or femoral neck BMD were defined as having low BMD.^[26]^ The BMD levels (gm/cm^2^) of the lumbar spine, total femur, femoral neck, trochanteric, and intertrochanteric region were measured using the dual-energy X-ray absorptiometry method by a Hologic QDR 4500A fan-beam densitometer (Hologic Inc., Bedford).^[27]^ *T*-score is calculated as: *T*-score = [BMD (measured) – mean BMD (reference)]/standard deviation (reference). The mean BMD (reference) was calculated based on BMD for non-Hispanic White women aged 20 to 29 years in the NHANES database.

### 2.3. Measurement of HGI and DAQS

HGI was used to reflect the difference between the measured HbA1c level and the predicted HbA1c level [HGI = HbA1c (measured) - HbA1c (predicted)].^[28]^ In this study, HGI was categorized into 2 groups based on median value (<−0.0835, ≥−0.0835).

DAQS was calculated based on 6 dietary antioxidant micronutrients, including vitamin A, vitamin C, vitamin E, zinc, magnesium, and selenium.^[25]^ For each dietary antioxidant, scores of 0 and 1 refer to intake <2/3 of the respective daily recommended intake and intake ≥2/3 of the recommended intake, respectively.^[29]^ The sum of DAQS ranged from 0 to 6, with 1 to 2 categorized as low quality, 3 to 4 as medium quality, and 5 to 6 as high quality. In this study, DAQS was divided into 2 groups (<5, ≥5).

### 2.4. Covariates

Participant data were collected including age, race, family poverty-to-income ratio, education, smoking, physical activity, drinking, body mass index (BMI), hypertension, dyslipidemia, history of fracture, cardiovascular disease, CKD, anemia, glucocorticoids, anticoagulant, sex hormones, antidiabetic agent, and anti-osteoporosis agent.

### 2.5. Statistical analysis

Continuous data were reported as mean and standard error (SE) and categorical data were described as numbers and percentages (n [%]). Student *t* test was utilized to determine the differences for continuous data and the chi-square test for categorical data. Covariates with missing values (missing < 10%) were interpolated using the random forest multiple interpolation method. There was no statistically significant difference before and after missing value interpolation (Table S1, Supplemental Digital Content, https://links.lww.com/MD/Q113).

Confounders related to low BMD were identified using a weighted univariate logistic regression model, and covariates with *P* < .05 were adjusted for in the multivariate logistic regression model (Table S2, Supplemental Digital Content, https://links.lww.com/MD/Q113). The relationship between HGI and low BMD in postmenopausal women was assessed by logistic regression models and reported as odds ratio and 95% confidence interval. The association between HGI and low BMD at different DAQSs was further analyzed. In addition, subgroup analyses were performed based on age and fracture history subgroups. Statistical analyses were completed using R version 4.2.3 software (R Foundation for Statistical Computing, Vienna, Austria). Statistically significant was defined as a *P*-value < .05 (2-sided).

## 3. Results

### 3.1. Participant characteristics

**Figure 1.**
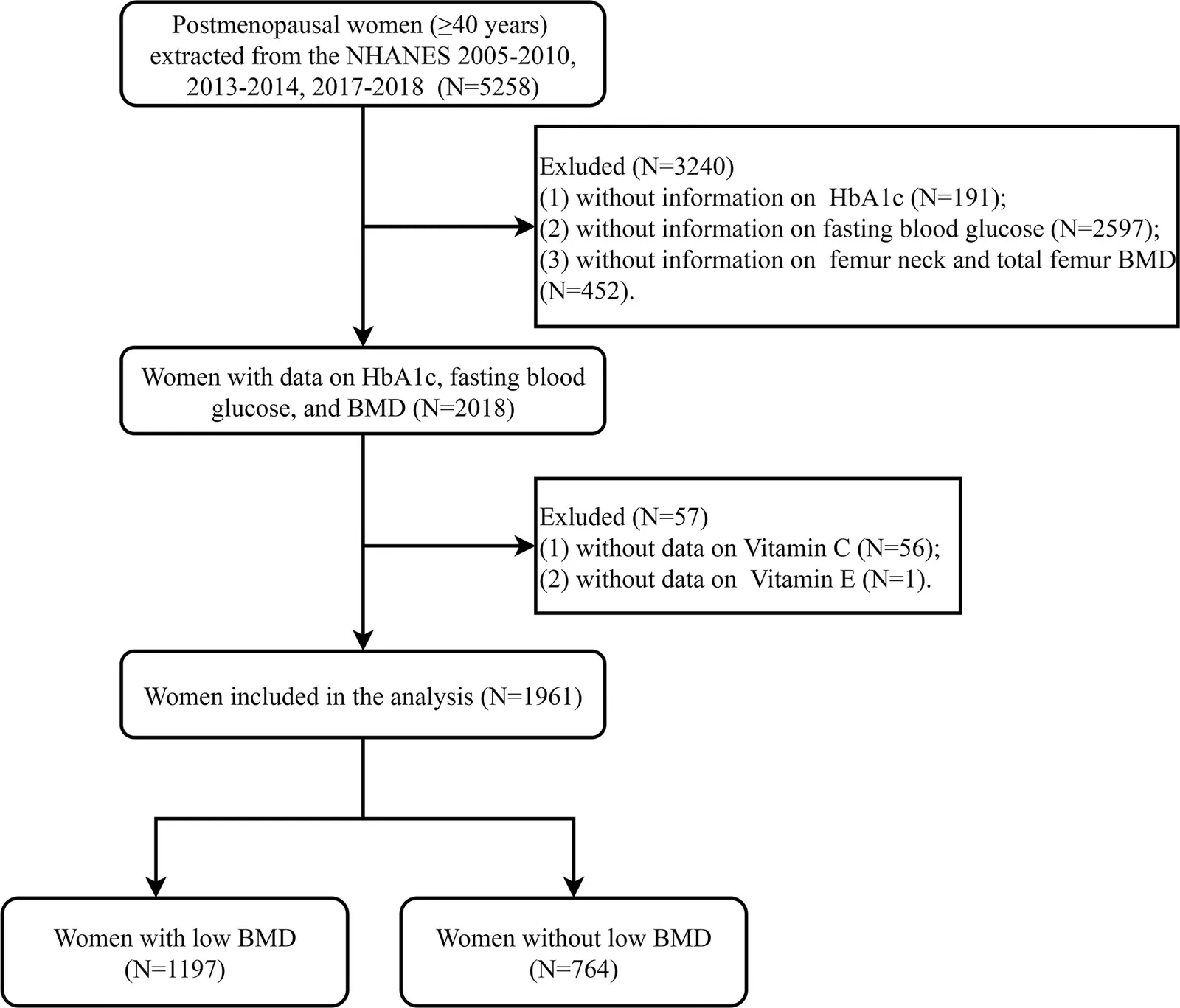
Screening flow chart of the study population.

A total of 5258 postmenopausal women from the NHANES database were screened, and 1961 eligible women were included in the final analysis (Fig. 1). The characteristics of women with and without low BMD were presented in Table 1. Of these 1961 women, 1065 (58.73%) were younger than 65 years of age and 896 (41.27%) were older than 65 years of age. The mean BMI was 28.51 (±0.19) kg/m^2^ and women with low BMD had a lower BMI. There were 280 (15.09%) women currently smoking, 923 (51.89%) women drinking, and 248 (13.75%) women with a history of fracture. There were 724 (42.14%) women with DAQSs ≥ 5 and 1237 (57.86%) with DAQSs < 5. Moreover, 1197 (60.56%) women had low BMD and 764 (39.44%) women did not have low BMD.

**Table 1:** Characteristics of postmenopausal women with and without low bone mineral density (BMD).

Variables	Total (n = 1961)	Non low BMD (n = 764)	Low BMD (n = 1197)	*P*
Age, n (%)				<.001
<65	1065 (58.73)	517 (72.61)	548 (49.69)	
≥65	896 (41.27)	247 (27.39)	649 (50.31)	
Race, n (%)				<.001
Mexican American	300 (5.38)	123 (5.63)	177 (5.22)	
Non-Hispanic Black	338 (8.69)	216 (14.74)	122 (4.75)	
Non-Hispanic White	997 (77.12)	325 (73.47)	672 (79.50)	
Other race	326 (8.81)	100 (6.16)	226 (10.53)	
PIR, n (%)				.458
<1	323 (9.84)	117 (9.19)	206 (10.27)	
≥1	1638 (90.16)	647 (90.81)	991 (89.73)	
Education, n (%)				.144
Above high school	910 (53.76)	374 (55.49)	536 (52.64)	
Below high school	533 (17.10)	187 (14.63)	346 (18.70)	
High school	518 (29.14)	203 (29.88)	315 (28.66)	
Smoking, n (%)				.578
Former	511 (28.51)	207 (28.81)	304 (28.32)	
Never	1170 (56.40)	449 (54.85)	721 (57.40)	
Now	280 (15.09)	108 (16.34)	172 (14.27)	
Physical activity, n (%)				.112
<750 MET·min/wk	1070 (49.36)	399 (46.50)	671 (51.22)	
≥750 MET min/wk	891 (50.64)	365 (53.50)	526 (48.78)	
Drinking, n (%)				.236
No	1038 (48.11)	388 (46.02)	650 (49.47)	
Yes	923 (51.89)	376 (53.98)	547 (50.53)	
BMI, kg/m^2^, mean (±SE)	28.51 (±0.19)	31.05 (±0.28)	26.85 (±0.20)	<.001
Hypertension, n (%)				.153
No	590 (35.80)	223 (32.84)	367 (37.73)	
Yes	1371 (64.20)	541 (67.16)	830 (62.27)	
Dyslipidemia, n (%)				.029
No	279 (13.76)	121 (16.52)	158 (11.96)	
Yes	1682 (86.24)	643 (83.48)	1039 (88.04)	
Fracture history, n (%)				.008
No	1713 (86.25)	700 (89.89)	1013 (83.88)	
Yes	248 (13.75)	64 (10.11)	184 (16.12)	
CVD, n (%)				.200
No	1580 (82.66)	634 (84.35)	946 (81.56)	
Yes	381 (17.34)	130 (15.65)	251 (18.44)	
CKD, n (%)				.139
No	1554 (82.87)	629 (84.56)	925 (81.76)	
Yes	407 (17.13)	135 (15.44)	272 (18.24)	
Anemia, n (%)				.286
No	1784 (93.57)	688 (92.90)	1096 (94.00)	
Yes	177 (6.43)	76 (7.10)	101 (6.00)	
Glucocorticoids, n (%)				.975
No	1915 (97.89)	746 (97.91)	1169 (97.88)	
Yes	46 (2.11)	18 (2.09)	28 (2.12)	
Anticoagulant, n (%)				.050
No	1915 (97.60)	753 (98.71)	1162 (96.88)	
Yes	46 (2.40)	11 (1.29)	35 (3.12)	
Sex hormones, n (%)				.125
No	1265 (61.63)	470 (58.84)	795 (63.44)	
Yes	696 (38.37)	294 (41.16)	402 (36.56)	
Anti-osteoporosis agent, n (%)				<.001
No	1834 (93.19)	750 (98.58)	1084 (89.68)	
Yes	127 (6.81)	14 (1.42)	113 (10.32)	
Antidiabetic agent, n (%)				.022
No	1636 (86.80)	621 (84.08)	1015 (88.56)	
Yes	325 (13.20)	143 (15.92)	182 (11.44)	
HGI, n (%)				.029
< −0.0835	855 (49.82)	327 (54.64)	528 (46.69)	
≥−0.0835	1106 (50.18)	437 (45.36)	669 (53.31)	
DAQS, n (%)				.314
0	46 (1.52)	20 (1.90)	26 (1.28)	
1	159 (7.62)	59 (7.00)	100 (8.03)	
2	217 (9.91)	73 (8.75)	144 (10.66)	
3	353 (16.33)	138 (18.28)	215 (15.07)	
4	462 (22.47)	200 (24.82)	262 (20.94)	
5	511 (28.88)	191 (26.61)	320 (30.36)	
6	213 (13.26)	83 (12.64)	130 (13.67)	

BMI = body mass index, CKD = chronic kidney disease, CVD = cardiovascular disease, DAQS = dietary antioxidant quality score, HGI = hemoglobin glycation index, PIR = family poverty-to-income ratio.

### 3.2. Relationship between HGI and low BMD in postmenopausal women

Compared with women with HGI < −0.0835, women with HGI ≥ −0.0835 (odds ratio [OR] = 1.38, 95% confidence interval [CI]: 1.03–1.83, *P* = .030) were correlated with higher odds of low BMD. After adjustment for age, race, education, BMI, fracture history, dyslipidemia, anti-osteoporosis agent, and antidiabetic agent, women with HGI ≥ −0.0835 (OR = 1.47, 95% CI: 1.09–1.98, *P* = .013) still had higher odds of low BMD.

Table 2 presents the relationship between HGI and low BMD at different DAQSs. Women with HGI ≥ −0.0835 were associated with higher odds of low BMD observed only in those with DAQSs <5 (OR = 1.44, 95% CI: 1.00–2.07, *P* = .049), while no association between HGI and low BMD was observed in those with DAQSs ≥5 (*P* = .150).

**Table 2:** The relationship between HGI and low BMD at different DAQSs.

Subgroups	Variables	Outcomes/Total	Low BMD	
			OR (95% CI)	*P*
DAQS < 5	HGI			
	<−0.0835	N = 313/516	Ref	
	≥−0.0835	N = 434/721	1.44 (1.00–2.07)	.049
DAQS ≥ 5	HGI			
	<−0.0835	N = 215/339	Ref	
	≥−0.0835	N = 235/385	1.42 (0.88–2.31)	.150

Multivariate logistic regression model adjusting for age, race, education, BMI, fracture history, dyslipidemia, anti-osteoporosis agent, and antidiabetic agent.

CI = confidence interval, DAQS = dietary antioxidant quality score, HGI = hemoglobin glycation index, OR = odds ratio, Ref = reference.

**Figure 2.**
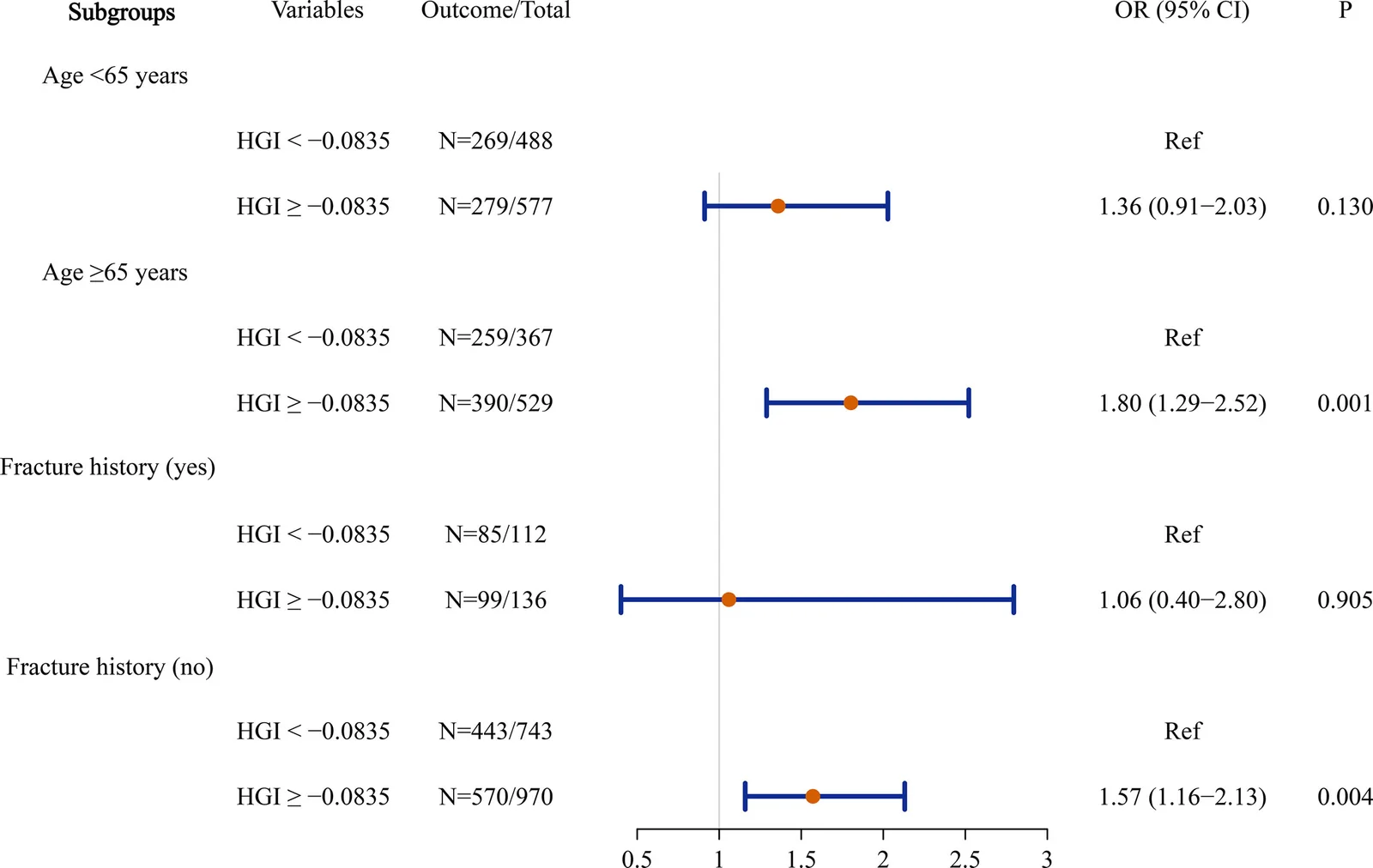
The association between hemoglobin glycation index (HGI) and low bone mineral density (BMD) in age and fracture history subgroups. BMD = bone mineral density, HGI = hemoglobin glycation index.

**Figure 3.**
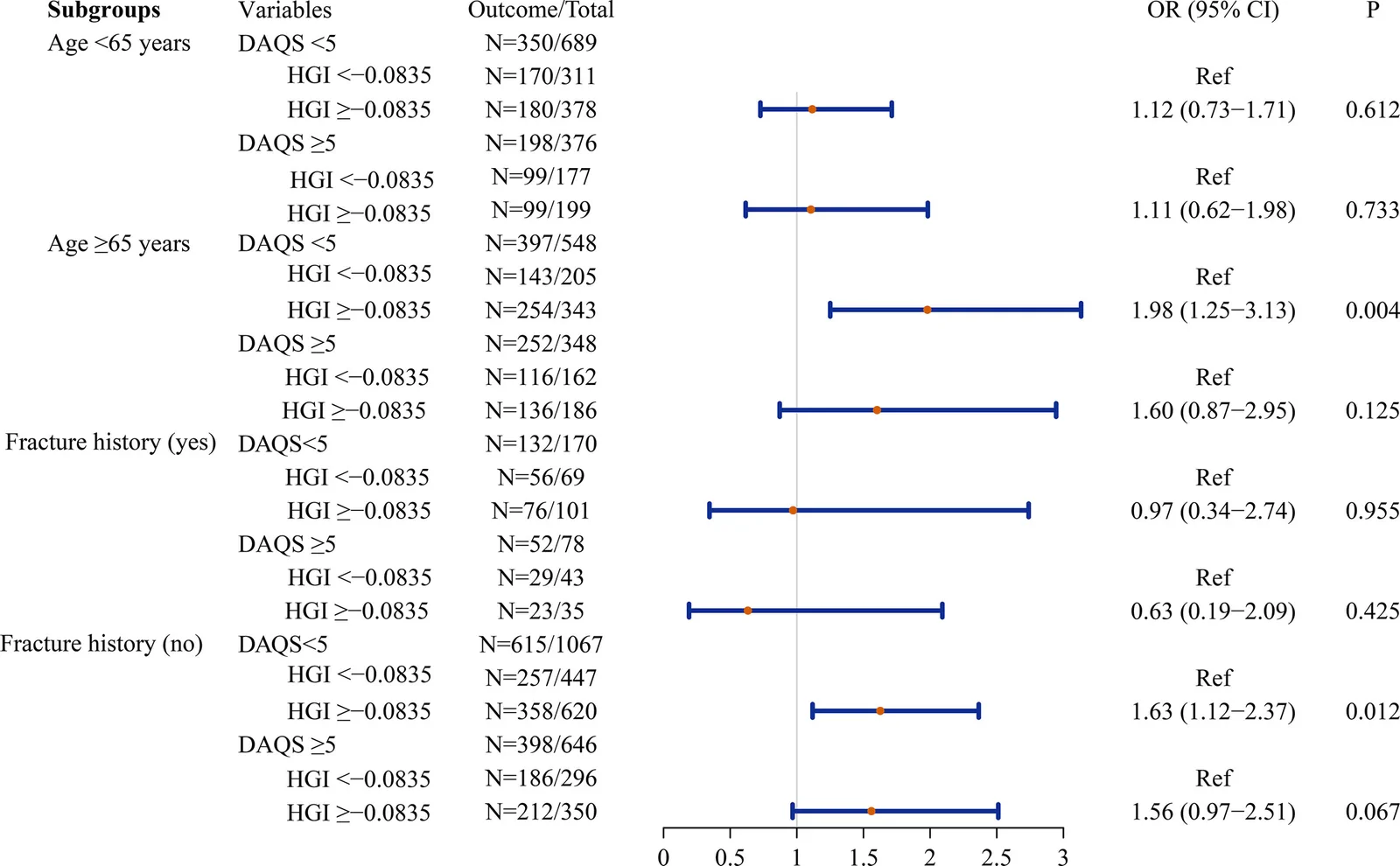
The relationship between HGI and low BMD in age and fracture history subgroups at different DAQSs. BMD = bone mineral density, DAQS = dietary antioxidant quality score, HGI = hemoglobin glycation index.

The association between HGI and low BMD in age and fracture history subgroups were presented in Figure 2. The relationship of HGI ≥ −0.0835 with higher odds of low BMD was observed only in women aged ≥65 years (OR = 1.80, 95% CI: 1.29–2.52), but not in women aged <65 years (*P* = .130). In addition, the correlation of HGI ≥ −0.0835 with higher odds of low BMD was found only in women without fracture history (OR = 1.57, 95% CI: 1.16–2.13), but not in women with fracture history (*P* = .905). The relationship between HGI and low BMD in age and fracture history subgroups at different DAQSs were shown in Figure 3. The relationships of HGI ≥ −0.0835 with higher odds of low BMD in the subgroups aged ≥65 years (OR = 1.98, 95% CI: 1.25–3.13) and without fracture history (OR = 1.63, 95% CI: 1.12–2.37) were found only in those with DAQSs < 5.

## 4. Discussion

This study analyzed the correlation between HGI and low BMD in postmenopausal women. High HGI was correlated with higher odds of low BMD in postmenopausal women, and this association was observed only in the subgroups aged ≥65 years or without fracture history. In addition, DAQSs may influence the relationship between high HGI and higher odds of low BMD, as this association was only observed in women with DAQSs < 5. Recent clinical evidence further validates our findings. Wen et al^[30]^ demonstrated that HGI directly reduces vertebral BMD (*r* = −0.140, *P* = .005) by enhancing osteoclast activity (β-CTX correlation: *r* = 0.15, *P* = .03), with 28.88% of bone loss mediated through this pathway. This aligns with our observed HGI-BMD association in postmenopausal women, particularly in the ≥65 age subgroup. Crucially, our study extends this mechanism by revealing that dietary antioxidants (quantified by DAQS) significantly attenuate HGI-induced bone damage an effect not captured in prior studies.

HbA1c levels can be used to indicate an individual’s mean blood glucose level over a period of 8 to 12 weeks, but the relationship between HbA1c levels and mean blood glucose varies among individuals.^[31,32]^ Hemoglobin glycosylation is another major factor besides blood glucose concentration that affects HbA1c levels, and HGI can be used to describe interindividual biological variation in HbA1c or the tendency of hemoglobin to be glycosylated.^[32,33]^ Elevated HGI may contribute to diabetic complications through inflammation and the formation of advanced glycosylation end products.^[34,35]^ Advanced glycosylation end products and oxidative stress produced by hyperglycemia may reduce enzyme beneficial cross-linking, inhibit osteoblast differentiation and induce osteoclast apoptosis.^[36]^ In nondiabetic patients, a high HGI is associated with higher fasting insulin levels and more severe insulin resistance.^[37]^ In this study, we analyzed the correlation between HGI and low BMD in postmenopausal women. The results indicated that high HGI was correlated with higher odds of low BMD, and this correlation was found in the subgroups aged ≥65 years or without fracture history. In addition, the association between HGI and low BMD may be affected by the DAQS since this association was only observed in women with DAQSs < 5. The DAQS is a composite of 6 dietary antioxidant intakes, with a high DAQS representing high antioxidant capacity. Some studies have shown that high DAQSs are associated with higher BMD.^[38,39]^ Antioxidant intake can reduce the production of free radicals and prevent the effects of oxidative damage on BMD.^[40,41]^

The relationship between HGI and BMD has not been reported, and only a few studies have reported the relationship between HbA1c and BMD. Higher HbA1c levels were associated with lower BMD in patients with type 1 diabetes.^[42]^ A Mendelian randomization study found that genetically increased HbA1c levels were associated with lower heel BMD.^[14]^ Bone loss increases with age and is even more pronounced in women than in men due to the loss of the protective effect of estrogen in women after menopause.^[43]^ Our results demonstrated that the association between high HGI and higher odds of low BMD was significant in women aged ≥65 years, but not in women aged <65 years. Moreover, our results showed that the relationship between HGI and low BMD was found only in women without fracture history, but not in women with fracture history. This may be because BMD is already damaged in women with fracture history and the effect of blood glucose on BMD is not significant. Moreover, the relationships of HGI with low BMD in the age and fracture history subgroups were also affected by the DAQS. The effect of dietary antioxidants on the relationship between HGI and low BMD may need to be explored in subsequent studies. Hou et al^[44]^ reported a paradoxical negative association between Hemoglobin A1c (HbA1c) levels and osteoporosis risk (odds ratio 0.914) within their general cohort. This finding contrasts with the consistent positive correlation between HGI and BMD observed in studies focused on dysglycemia, including our own and that of Wen et al This discrepancy underscores 2 key points: the superiority of HGI over HbA1c in predicting bone loss, as HGI accounts for individual variability in glycation, and the population-specific effects, as Hou et al’s cohort was predominantly nondiabetic, whereas our study focused on high-risk postmenopausal women. Furthermore, our findings regarding the interaction with dietary antioxidant quality score (DAQS; association observed only at DAQS < 5) suggest that antioxidant intake modulates the glycotoxicity mechanisms described in both studies.

In this study, the relationship between HGI and low BMD in postmenopausal women was analyzed for the first time. HGI can be more accurately used to present the association between blood glucose levels and low BMD than HbA1c and fasting blood glucose. Moreover, the relationship between HGI and low BMD may be influenced by dietary antioxidants. Nevertheless, several limitations of this study should be noted. First, the analysis of the relationship between HGI, low BMD, and DAQS based on data from cross-sectional surveys could not confirm chronological and causal associations due to the limitations of the NHANES database. Second, although the study included potential confounders such as demographics, socioeconomics, comorbidities, and medication use to the extent possible, there may still be other confounders (e.g., detailed treatment information) that could affect the results.

## 5. Conclusions

High HGI was associated with higher odds of low BMD in postmenopausal women, and this relationship was found in women aged ≥65 years or without fracture history. Moreover, the association between high HGI and higher odds of low BMD was influenced by the DAQS. Subsequent studies need to assess the effect of dietary antioxidants on the relationship between HGI and low BMD.

## Author contributions

**Conceptualization:** Yuchen Wang.

**Data curation:** Chuan Jia.

**Investigation:** Wenke Zhu.

**Methodology:** Wenke Zhu, Changjun Yun.

**Software:** Chuan Jia.

**Validation:** Changjun Yun.

**Writing – original draft:** Yuchen Wang.

**Writing – review & editing:** Yuchen Wang, Weizhong Yu.

## Supplementary Material
