# Supplementary material for: Association between hemoglobin glycation index and low bone mineral density in postmenopausal women: An analysis of data from the NHANES database
Source: Medicine (Baltimore). 2025 Sep 26;104(39):e44771. doi: 10.1097/MD.0000000000044771 (PMC13593182; doi:10.1097/MD.0000000000044771)
Supplement: Supplementary file 1 [file medi-104-e44771-s001.pdf]

Supplement Table 1. Difference analysis before and after missing value interpolation.

Variables	Before interpolation (N=1961)	After interpolation (N=1961)	Statistics	*P*
Fracture, n (%)			χ^2 = 1.757	0.189
No	1711 (86.24)	1713 (86.25)		
Yes	248 (13.76)	248 (13.75)		
Education, n (%)			χ^2 = 2.142	0.144
Above high school	530 (17.08)	533 (17.10)		
Below high school	518 (29.15)	518 (29.14)		
High school	909 (53.77)	910 (53.76)		
Hemoglobin, Mean (±S.E)	13.76 (±0.05)	13.76 (±0.05)	t = -0.033	0.974
Anemia, n (%)			χ^2 = 2.917	0.092
No	1780 (93.56)	1784 (93.57)		
Yes	177 (6.44)	177 (6.43)		
Height, Mean (±S.E)	160.88 (±0.22)	160.87 (±0.22)	t = -1.269	0.208
Weight, Mean (±S.E)	73.87 (±0.52)	73.87 (±0.52)	t = -0.562	0.576
Sex hormones, n (%)			χ^2 = 0.143	0.707
Yes	693 (38.38)	696 (38.37)		
No	1260 (61.62)	1265 (61.63)		
Physical activity, n (%)			χ^2 = 1.727	0.193
<750 MET· min/week	994 (48.86)	1070 (49.36)		
≥750 MET· min/week	836 (51.14)	891 (50.64)		
PIR, n (%)			χ^2 = 0.743	0.391
<1	295 (9.98)	323 (9.84)		
≥1	1496 (90.02)	1638 (90.16)		

Note: PIR, family poverty-to-income ratio.

Supplement Table 2. Identification confounders related to low BMD using a weighted univariate logistic regression model.

Variables	Outcome/Total	OR (95% CI)	*P*
Age			
<65	N=548/1065	Ref	
≥65	N=649/896	2.68 (2.04-3.53)	<0.001
Race			
Mexican American	N=177/300	Ref	
Non-Hispanic Black	N=122/338	0.35 (0.24-0.50)	<0.001
Non-Hispanic White	N=672/997	1.17 (0.86-1.58)	0.316
Other Race	N=226/326	1.84 (1.26-2.69)	0.002
PIR			
<1	N=206/323	Ref	
≥1	N=991/1638	0.88 (0.64-1.23)	0.459
Education			
Above High School	N=536/910	Ref	
Below High School	N=346/533	1.35 (1.02-1.79)	0.039
High School	N=315/518	1.01 (0.78-1.32)	0.934
Smoking			
Former	N=304/511	Ref	
Never	N=721/1170	1.06 (0.78-1.45)	0.687
Now	N=172/280	0.89 (0.63-1.26)	0.506
Drinking			
No	N=650/1038	Ref	
Yes	N=547/923	0.87 (0.69-1.10)	0.236
Physical activity			
<750	N=671/1070	Ref	
≥750	N=526/891	0.83 (0.65-1.05)	0.112
BMI	N=1197/1961	0.88 (0.86-0.91)	<0.001
Hypertension			
No	N=367/590	Ref	
Yes	N=830/1371	0.81 (0.60-1.08)	0.153
Anemia			
No	N=1096/1784	Ref	
Yes	N=101/177	0.83 (0.60-1.17)	0.287
Dyslipidemia			
No	N=158/279	Ref	
Yes	N=1039/1682	1.46 (1.04-2.04)	0.030
Fracture history			
No	N=1013/1713	Ref	
Yes	N=184/248	1.71 (1.15-2.53)	0.008

Variables	Outcome/Total	OR (95% CI)	*P*
CVD			
No	N=946/1580	Ref	
Yes	N=251/381	1.22 (0.90-1.65)	0.200
CKD			
No	N=925/1554	Ref	
Yes	N=272/407	1.22 (0.94-1.60)	0.140
Glucocorticoids			
No	N=1169/1915	Ref	
Yes	N=28/46	1.01 (0.45-2.28)	0.975
Anticoagulant			
No	N=1162/1915	Ref	
Yes	N=35/46	2.46 (0.97-6.25)	0.057
Sex hormones			
No	N=795/1265	Ref	
Yes	N=402/696	0.82 (0.64-1.06)	0.125
Anti-osteoporosis agent			
No	N=1084/1834	Ref	
Yes	N=113/127	7.97 (3.60-17.62)	<0.001
Antidiabetic agent			
No	N=1015/1636	Ref	
Yes	N=182/325	0.68 (0.49-0.95)	0.023

Note: PIR, family poverty-to-income ratio; BMI, body mass index; CVD, cardiovascular disease; CKD, chronic kidney disease; OR, odds ratio; CI, confidence interval; Ref, reference.
